# Compensation through Functional Hyperconnectivity: A Longitudinal Connectome Assessment of Mild Traumatic Brain Injury

**DOI:** 10.1155/2016/4072402

**Published:** 2015-12-27

**Authors:** Armin Iraji, Hanbo Chen, Natalie Wiseman, Robert D. Welch, Brian J. O'Neil, E. Mark Haacke, Tianming Liu, Zhifeng Kou

**Affiliations:** ^1^Department of Biomedical Engineering, Wayne State University, Detroit, MI, USA; ^2^Department of Computer Science, The University of Georgia, Athens, GA, USA; ^3^Department of Psychiatry and Behavioral Neurosciences, Wayne State University, Detroit, MI, USA; ^4^Department of Emergency Medicine, Wayne State University, Detroit, MI, USA; ^5^Department of Radiology, Wayne State University, Detroit, MI, USA

## Abstract

Mild traumatic brain injury (mTBI) is a major public health concern. Functional MRI has reported alterations in several brain networks following mTBI. However, the connectome-scale brain network changes are still unknown. In this study, sixteen mTBI patients were prospectively recruited from an emergency department and followed up at 4–6 weeks after injury. Twenty-four healthy controls were also scanned twice with the same time interval. Three hundred fifty-eight brain landmarks that preserve structural and functional correspondence of brain networks across individuals were used to investigate longitudinal brain connectivity. Network-based statistic (NBS) analysis did not find significant difference in the group-by-time interaction and time effects. However, 258 functional pairs show group differences in which mTBI patients have higher functional connectivity. Meta-analysis showed that “Action” and “Cognition” are the most affected functional domains. Categorization of connectomic signatures using multiview group-wise cluster analysis identified two patterns of functional hyperconnectivity among mTBI patients: (I) between the posterior cingulate cortex and the association areas of the brain and (II) between the occipital and the frontal lobes of the brain. Our results demonstrate that brain concussion renders connectome-scale brain network connectivity changes, and the brain tends to be hyperactivated to compensate the pathophysiological disturbances.

## 1. Introduction

Mild traumatic brain injury (mTBI) represents a major public health burden with an annual incidence of over 1.2 million people in the USA [1–3]. Despite normal findings in clinical imaging such as computed tomography (CT) and structural magnetic resonance imaging (MRI) for the majority of mTBI patients, mTBI can cause emotional, physical, and cognitive symptoms that significantly impact the patients' quality of life and cost the nation $16.7 billion each year [4–6]. TBI in general can be thought of as a disorder of network disconnection [7]. Similar clinical presentations are observed in diverse range of insult and injury despite different origins and locations of impact. Therefore, evaluating the outcome of injury in specific brain regions is confusing, and it provides a distorted view of brain functional disruption [8]. This situation is more significant in mild TBI than in severe and moderate TBI because there is usually no significant structural damage at mild TBI, indicating that this may account for a greater portion of the problem in mTBI than in moderate or severe TBI [9].

In light of heterogeneous locations of injury, use of large-scale approaches can provide a better understanding of brain function and improve neuropsychological understanding of the sequela of mTBI. Furthermore, mapping the topography of the brain functional connectivity alterations at a macrolevel can elucidate the role of brain plasticity. It has been reported that brain injury induces not only structural damage, which could be detected by diffusion tensor imaging (DTI), but also functional disturbances and compensation, which are considered a neural plastic effect [10–13]. In adults, brain plasticity still takes place, especially at a large scale, which is known as adult plasticity [9, 14, 15]. In general, brain plasticity can be defined as the capacity of the brain to change its structural or functional connectivity and organization during a short or long period of time [16, 17]. In adult plasticity, according to the Hebbian theory, coincident neuronal firing leads to wiring together [18]. At the same time, brain functional connectivity is measured using the temporal dependency of neural activity [19]. Therefore, it has been suggested that functional connectivity (FC) of the brain during the resting state is linked to the brain plasticity [20], which has been confirmed by showing a strong correlation between brain plasticity and resting-state FC (rs-FC) changes [21, 22].

Accumulating evidences demonstrate brain plasticity after brain injury. Zhou et al. [20] studied brain plasticity using rs-FC after partial and complete callosotomy groups, in which disrupted functional connectivity was restored at day 28 in the partial callosotomy group through the remaining interhemispheric axonal pathways. Additionally, intrahemispheric functional connectivity was increased in both partial and complete callosotomy, representing plasticity in brain FC and adaptation after injury [20]. Nakamura et al. [23] examined neural plasticity in a longitudinal study during recovery period of TBI using graph theory, which revealed that overall functional connectivity strength is higher at three months after injury as compared with what is observed in healthy adults; however, functional connectivity decreases and approaches that of the healthy subjects sample after six months after injury. These findings indicate that overall functional connectivity increases to compensate the effect of injury after TBI [23]. Therefore, the study of neuroplasticity can provide a new angle to inspect the neurological origins of brain alterations after injury, especially at a large scale, and provide new insights into the pathophysiological substrates of cognitive and behavioral alterations [7, 24]. However, to date, the field is still short of investigation on connectome-scale brain network alterations and compensatory effects after TBI. Moreover, the impacts of brain alterations over time in connectome-scale brain networks during brain recovery are still unknown [11]. We hypothesize that mTBI induces brain network disturbances and also the consequent compensatory effects at the connectome-scale network level. Our objective is to investigate the connectome-scale brain network changes after mTBI and recovery and potential brain plasticity.

Resting-state functional MRI (rsfMRI) constitutes an ideal way to evaluate brain functional connectivity and changes in brain functional connectivity at a connectome scale which can contribute to a better understanding of plasticity and the brain's attempts to compensate for injury [7–9]. Brain functional connectivity can be measured using statistical relationships between the blood-oxygen-level dependent (BOLD) signals of brain regions, which reflects the prior history of coactivation between brain regions [25]. The large-scale rs-FC is an ideal way to evaluate brain functional connectivity on a connectome scale at a macrolevel, which can be used to investigate brain plasticity and serve as an index of the efficacy of healing [8]. rsfMRI provides unique insights into brain plasticity at the macrolevel, which can provide a better understanding of brain disorders such as TBI and consequently guide proper rehabilitation plan [11].

In the evaluation of connectome-scale brain networks, one technical challenge is to define the network nodes or regions of interest (ROIs). The network nodes serve as structural and functional substrates for network analysis. Several approaches are available in the field with remarkable progress. However, they also suffer different limitations from different perspectives, particularly at a large scale, when the network is at very fine level. Any mismatch will result in the cross talk of a network to its neighbors. Zhu et al. previously identified 358 regions of interest (ROIs), known as dense individualized common connectivity based cortical landmarks (DICCCOLs), distributed across the brain. The location of each DICCCOL is identified based on the white matter fiber connection profile obtained from diffusion MRI (dMRI). Each DICCCOL preserves similar structural and functional properties across individuals [26]. Since each DICCCOL preserves consistent structural connection patterns, according to the connectional fingerprint concept, its functional role should be similar across subjects [27]. In addition to reproducibility and consistency, DICCCOL analysis has been shown to be powerful in identifying connectivity signatures (disrupted connectivity) in affected brains [28]. After localizing DICCCOLs across an individual's brain, the time series related to each DICCCOL is derived from the BOLD rsfMRI signal of the gray matter area in the 5 mm neighborhood of each DICCCOL. The functional connectivity of an individual's brain at a large scale is assessed by measuring temporal Pearson correlation between each pair of the time series allocated to the DICCCOLs. Our goal is to use the DICCCOL framework to define the functional network nodes at a large scale and further assess the effect of brain injury on mTBI patients over time.

## 2. Materials and Methods

### 2.1. Data Acquisition

Patient eligibility was based on the mTBI definition by the American Congress of Rehabilitation Medicine, 1993, with the following inclusion criteria: patients aged 18 or older with an initial Glasgow Coma Scale (GCS) score of 13–15 in the ED with any loss of consciousness less than 30 minutes or any posttraumatic amnesia less than 24 hours, or recorded change of mental status (confused, disoriented, or dazed). In the acute stage (first scan), patients were scanned at 82.64/17 hours (average/median) after injury, and the second scan was acquired at 4–6 weeks after injury (subacute stage). For healthy subjects, two scans were acquired with a 4–6-week interval in between. rsfMRI and dMRI data were acquired on a 3-Tesla Siemens Verio scanner with a 32-channel radiofrequency head-only coil. Demographic characteristics are presented in Table 1. dMRI data has been already used and analyzed for another study. Thus, dMRI has only been used to identify the locations of DICCCOLs in this study. dMRI data was acquired using a gradient echo EPI sequence with *b* = 0/1000 s/mm^2^ in 30 diffusion gradients directions with the following parameters: TR (repetition time) = 13300 ms, TE (echo time) = 124 ms, slice thickness = 2 mm, pixel spacing size = 1.333 × 1.333 mm, matrix size = 192 × 192, flip angle = 90°, and number of averages (NEX) = 2. For rsfMRI data, gradient echo EPI sequence with following imaging parameters has been performed: pixel spacing size = 3.125 × 3.125 mm, slice thickness = 3.5 mm, slice gap = 0.595 mm, matrix size = 64 × 64, TR/TE = 2000/30 ms, flip angle = 90°, 240 volumes for whole-brain coverage, and NEX = 1. During rsfMRI scans, subjects were instructed to keep their eyes closed and not to think about anything specific. In addition, T1, T2, fluid attenuated inversion recovery (FLAIR), and susceptibility weighted imaging (SWI) were also acquired. The total data acquisition protocol took about 40 minutes.

### 2.2. Data Analysis

A series of steps were taken to analyze the data: (1) localizing DICCCOL nodes of each subject using the DICCCOL framework and computing FC between the time series of each pair of DICCCOLs; (2) performing a longitudinal statistical analysis using mixed 2 × 2 design analysis of variance (ANOVA) and network-based statistic (NBS) analyses to determine the connectomic signatures; (3) determining the functional roles of the connectomic signatures using meta-analysis; and (4) performing multiview group-wise cluster analysis to determine involved FC clusters and the interrelationships of the connectomic signatures.

#### 2.2.1. Localizing DICCCOLs and Computing FC Matrix for Each Subject

**Figure 1:**
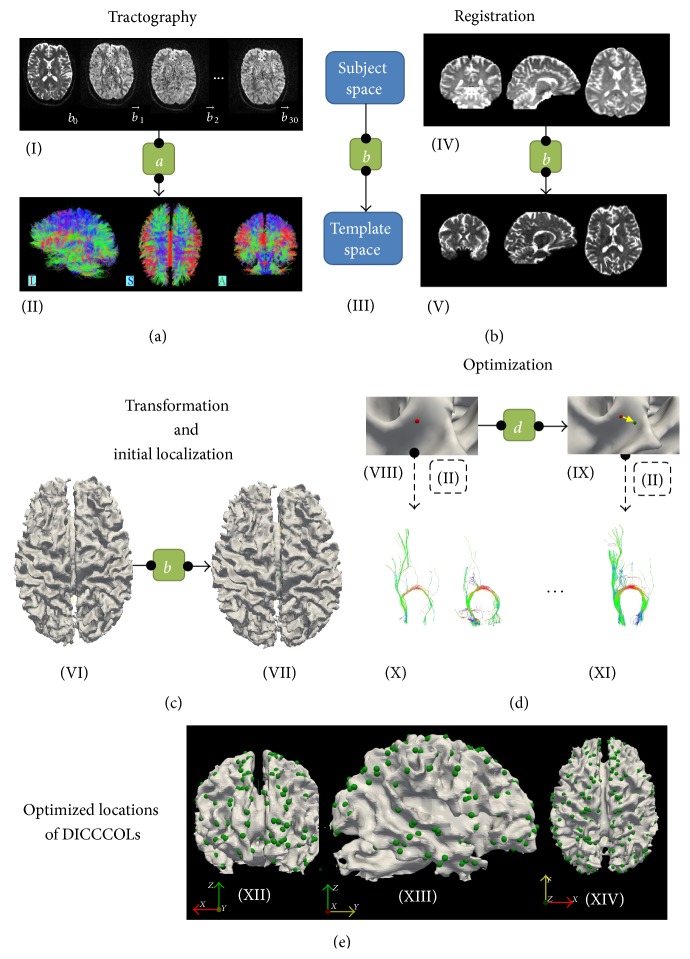
Pipeline for identifying the locations of DICCCOLs on the brain of an individual. (a) Fiber tracking and tractography of the whole brain was performed via MedINRIA (http://med.inria.fr/). Box “*a*” presents the preprocessing steps (including brain extraction, motion correction, and eddy current correction) and deterministic tractography. (a)(I) shows diffusion data of an individual brain at *b*_0_ and some different gradient directions, and (a)(II) shows the result of tractography in 3D space in the sagittal, axial, and coronal views. (b) The transformation matrix to transfer coordinates from the subject space to the template space was obtained by registering the brain of an individual subject to the brain template. (b)(III) shows the schematic of this procedure in which box *b* represents the transformation matrix. (b)(IV) and (b)(V) show the coronal, axial, and sagittal views of individual and template's brains, respectively. (c) Transformation and identification of the initial location of DICCCOLs. The transformation matrix (*b*) is applied to transfer the individual surface and fiber bundles to the template space for prediction. As a result, the initial location of DICCCOLs on the individual's brain is obtained. (c)(VI) is the surface of an individual in the subject space, and (c)(VII) is the surface of the same individual, which is transferred to the template space. The initial location of DICCCOLs on an individual's brain was obtained by overlaying the location of DICCCOLs of the template on the transformed surface of the individual. (d) The schematic procedure of optimization in which the local neighborhood (6 mm radius) was searched in order to identify the location where the profile of connected fiber has the most similarity with the WM fiber connection profile of the DICCCOL on the template. (d)(VIII) shows the initial location of a DICCCOL, obtained from the previous step. Using the information of deterministic tractography ((a)(II)), the connected fiber at this initial location was extracted ((d)(X)). Next, the similarity between the connected fibers at this location and the connected WM fiber on the template was measured. The same procedure took place for all local neighborhoods, and the location with maximum similarity of the connected WM fibers was identified as the optimized location of the DICCCOL. Box *d* represents the optimization procedure. (d)(IX) shows the initial and optimized locations of a DICCCOL in red and green, respectively. (d)(X) and (d)(XI) show the connected fibers at the initial and optimized locations of the DICCCOL, respectively. (e) represents the optimized locations of all DICCCOLs on an individual's brain. (e)(XII), (e)(XIII), and (e)(XIV) show the coronal, sagittal, and axial views in 3D space, respectively.

In the connectomic study, one essential step is to identify the locations of ROIs across the brain that preserve consistent structural and functional connections across individuals. DICCCOLs are a set of brain landmarks with similar structural and functional connectivity across individuals obtained by identifying the consistent white matter (WM) fiber connection profile across subjects. This has been done using the tool available to download at http://dicccol.cs.uga.edu/. Identifying the locations of DICCCOLs on the brain of an individual can be summarized in the following steps (see Figure 1): (1) deterministic tractography of whole brain was performed (Figure 1(a)). As a result, we were able to identify the WM fiber connection profile of each location of the brain. (2) Extraction of the transformation matrix was performed for the coregistering of the brain of an individual subject to the brain template (Figure 1(b)). (3) Application of the transformation matrix was carried out to transfer the individual surface and fiber bundles to the template space for prediction (Figure 1(c)). As a result, the initial location of DICCCOLs on the individual's brain was obtained. (4) Searching the local neighborhood (6 mm radius) of the initial location of each DICCCOL was conducted to find the optimized location of that DICCCOL. For this purpose, similarity between the WM fiber connection profile in the template and all local neighborhoods was measured. The neighborhood with the maximum similarity of the WM fiber connection profile with the WM fiber connection profile of the DICCCOL on the template was selected as the optimized location of the DICCCOL (Figure 1(d)). This was performed to identify optimized locations of all DICCCOLs on each individual's brain (Figure 1(e)). DICCCOL landmarks have been shown to be highly reproducible across individuals [28]. At the same time, according to the connectional fingerprint concept, each brain's cytoarchitectonic area has a unique set of extrinsic inputs and outputs that largely determines the functions that each brain area performs [27]. The close relationships between structural connection patterns and brain functions have extensively been reported [29–31]. Since each DICCCOL preserves consistent structural connection patterns, its functional role should be similar across subjects. The intrinsic functional role of each DICCCOL has been already extensively examined and validated [28, 32]. On the other hand, although function-based ROIs are helpful for wide range of studies, they seem to not be an appropriate choice for connectome studies, which require fine identification of ROI locations, because the accuracy of ROIs locations in function-based ROIs can be compromised by several factors such as normalization and spatial smoothing [30]. Therefore, the DICCCOL framework was applied to identify the corresponding ROIs across individuals for FC analysis.

**Figure 2:**
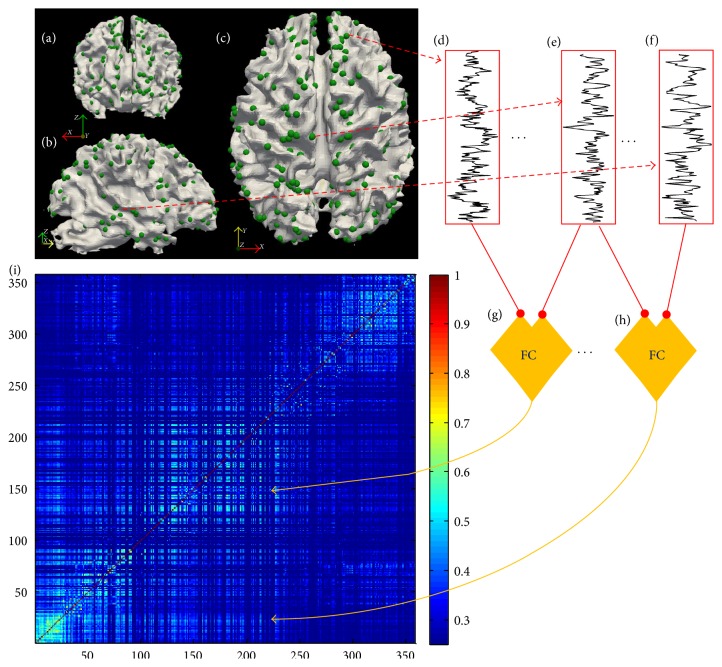
Functional connectivity (FC) was measured across the brain using the DICCCOL framework. (a), (b), and (c) show the optimized locations of DICCCOLs across the brain in the coronal, sagittal, and axial views, respectively. (d), (e), and (f) represent the time series allocated to three DICCCOLs obtained from the rsfMRI data of the gray matter of the 5 mm neighborhood of each DICCCOL. The Pearson correlation was calculated between time series of 358 DICCCOLs in order to obtain the FC of the brain at large scale. (g) and (h) show two examples of FC measurement, and (i) is a symmetric affinity matrix, which represents FC at a connectomic level.

For FC analysis of the rsfMRI data using FSL, preprocessing included brain extraction, motion correction, slice-time correction, spatial smoothing (FWHM = 5 mm), temporal prewhitening, grand mean removal, and temporal high-pass filtering [33]. The time series allocated to each DICCCOL was derived from the gray matter area in the 5 mm neighborhood of that DICCCOL. FC between each pair of DICCCOLs was obtained by measuring temporal Pearson correlations between the time series allocated to each DICCCOL. Therefore, a symmetric affinity matrix with 64261 unique features was obtained to represent functional connectivity of the brain at a connectomic level (Figure 2).

#### 2.2.2. Network-Based Statistical (NBS) Analysis

Neuroimaging studies suggest that networks compose cognitive and functional domains of the brain. At the same time, the networks for different cognitive and functional domains can overlap with each other [34]. To fully investigate the disrupted cognitive and functional domains of the brain, meta-analysis and multiview group-wise clustering approaches were applied. For the statistical longitudinal analysis, a mixed 2 × 2 design ANOVA was used to identify connectomic signatures. Independent variables were group (controls versus patients) and time (acute versus subacute). NBS is a validated method which was originally developed for connectomic studies to perform nonparametric statistical analysis on large-scale pairs of connections [35]. While the false discovery rate (FDR) is sensitive to detecting an independent, isolated connected pair regardless of its affiliation (or conjunction) with other connected pairs, NBS improves power to detect a nexus that includes multiple affiliated connected pairs [35]. In other words, NBS, while controlling the family-wise error rate (FWER), implements rejection of a null hypothesis at the network level. NBS, intuitively, includes the following steps:

(a) Performing a statistical test on each connected pair independently. NBS, like other common neuroimaging software packages, uses the general linear model (GLM) as the statistical test. The output of this step is a set of connected pairs that are the potential candidates to be connectomic signatures (connected pairs which are statistically different between two groups).
(b) Identifying any possible connectivity structure from connected pairs which were selected at the previous step.
(c) Calculating a FWER-corrected *p* value for a connectivity structure with size of K using permutation testing. Specifically, for each permutation, connectivity structures were identified and the maximal component size was obtained. Then, *p* value for any observed connectivity structure with size of K can be calculated based on the possibility of having maximal connectivity structure size > K in M permutation.

NBS parameters, including the uncorrected threshold value, were chosen as follows according to previous published literature [36–39]: threshold = 3.5, permutation = 5000, component size = extent, and *p* value = 0.05. For statistical longitudinal analysis, a mixed 2 × 2 design ANOVA has been used to identify connectomic signatures. Independent variables are group (controls versus patients) and time (acute versus subacute). If the interaction effect was not significant, we investigated the group and time effects. Otherwise, we investigated each simple effect. In other words, if the interaction effect was significant, we used a two-sample unpaired *t*-test to compare two groups and a two-sample paired *t*-test to investigate each group over time.

#### 2.2.3. Categorizing Connectomic Signatures Using Meta-Analysis

BrainMap (http://www.nitrc.org/projects/brainmap/) is an online database of published neuroimaging literature, which can be used to identify the function of brain regions based on previous reported data from well-credited labs [40]. Yuan et al. used the BrainMap software to identify the possible functional roles for each DICCCOL using meta-analysis [32]. Using the BrainMap database, we can categorize the DICCCOLs involved in connectomic signatures into five general functional categories, including “Action,” “Perception,” “Cognition,” “Interoception,” and “Emotion,” and these five categories can be further divided into 53 subcategories. At the same time, the strength of functional connectivity between two DICCCOLs can be part of the strength of functional connectivity between their corresponding networks. For instance, if DICCCOL “A” was identified as “Cognition” and DICCCOL “B” was identified as “Emotion,” then we can interpret that the strength of functional connectivity between “A” and “B” is related to the strength of functional connectivity between “Cognition” and “Emotion.” Therefore, by categorizing the DICCCOLs involved in connectomic signatures to these functional categories, we can have better understanding of brain functional and cognitive interactions at large scale.

#### 2.2.4. Multiview Group-Wise Cluster Analysis

Multiview group-wise clustering has been applied to the DICCCOL system to extract the substrate brain functional clusters as previously described by Chen et al. [41]. A cluster is defined as a set of DICCCOLs that have stronger inner functional connectivity with each other rather than with the DICCCOLs of other clusters. If we consider the DICCCOL system to be a graph representation of the brain, the DICCCOLs can be considered to be the nodes of the graph, and functional connectivities between DICCCOLs are the edges of the graph. For each subject, the functional connectivity matrix of the brain was extracted and considered to be one “view” for the clustering approach. The group-wise clustering method was applied to categorize common clusters across individuals. Briefly, in this clustering method, the clusters were obtained by projecting each view on the others to derive consistent functional clusters across brain functional connectivity of individuals [41]. After identifying the brain functional clusters, the connectomic signatures of mTBI were categorized into these identified clusters to reveal possible patterns in functional connectivity alterations of the brain after injury.

## 3. Results 

**Figure 3:**
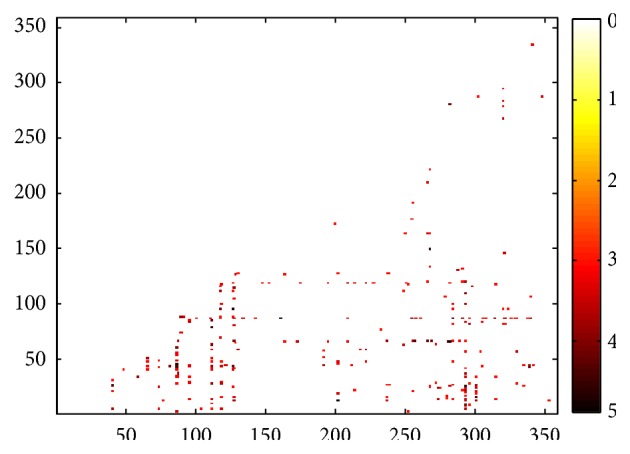
*T*-values from the longitudinal statistical analysis using a mixed design ANOVA and NBS on the 258 FC pairs which are significantly stronger in the patient group. Since the FC matrices are symmetric, only the lower half was used for the statistical analysis.

Statistical analysis (Section 2.2.2) was performed on the brain functional connectivity, which was obtained from 24 healthy subjects and 16 mTBI patients at two time points. No statistically significant difference was found in the interaction effect (*p* value = 0.05). The time effect did not show any significant difference (*p* value = 0.05), either. However, in our NBS analysis, which only considers the interconnected network clusters, we identified a group effect on 258 FC pairs that were significantly affected in mTBI patients (Figure 3). All of these affected FC pairs (i.e., connectomic signatures) showed increased functional connectivity in the patient group.

**Figure 4:**
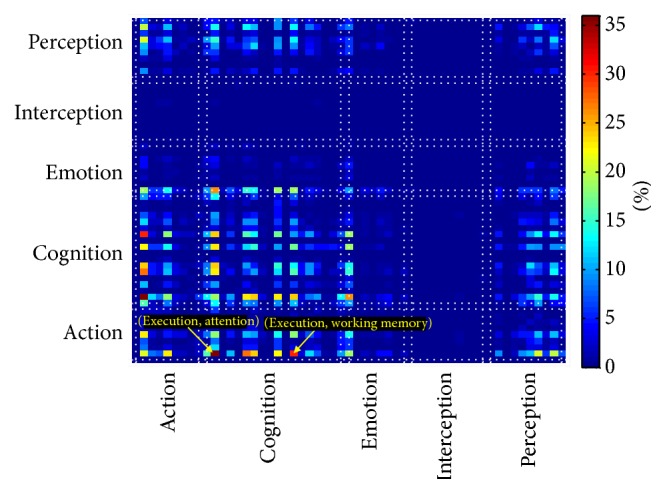
Categorization of altered functional connectivity in mTBI. The color-bar indicates percent change. The results show that the “Action” and “Cognition” networks have been disrupted the most. Further functional analysis using the 53 subcategories shows that interaction between “Execution” (from the “Action” category) and “Attention” (from the “Cognition” category) and between “Execution” (from the “Action” category) and “Working Memory” (from the “Cognition” category) has been affected the most (yellow arrow). This is consistent with published literature on Attention, Working Memory, and executive function deficits in mTBI patients.

In order to have a better understanding of changes in brain function after mTBI, meta-analysis was performed. The affected functional connectivity was categorized into five major brain functional domains: “Action,” “Perception,” “Cognition,” “Interoception,” and “Emotion.” We have observed that “Action” and “Cognition” are more involved in altered functional connectivity after mTBI, especially the interaction between “Action” and “Cognition” networks, which has been affected the most (Figure 4). Further investigation of the roles of DICCCOLs which were evaluated in greater detail using 53 subcategories reveals that the interactions between “Execution” (from the “Action” category) and “Attention” (from the “Cognition” category) and between “Execution” (from “Action” category), and “Working Memory” (from “Cognition” category) were affected the most (Figure 4).

**Figure 5:**
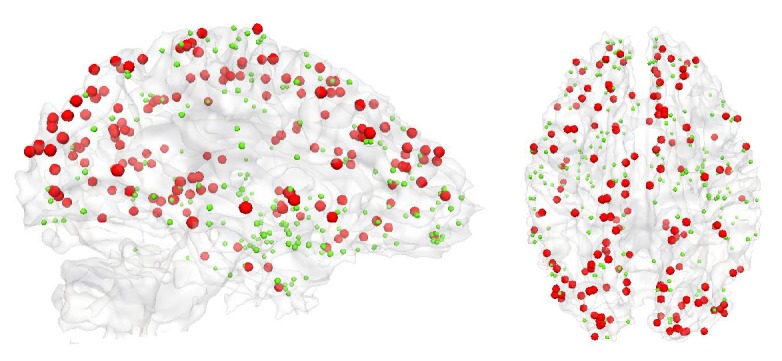
Visualization of location of DICCCOLs involved in affected functional connectivity signatures (red sphere) and the remaining DICCCOLs (green sphere) on cortical surface.

**Figure 6:**
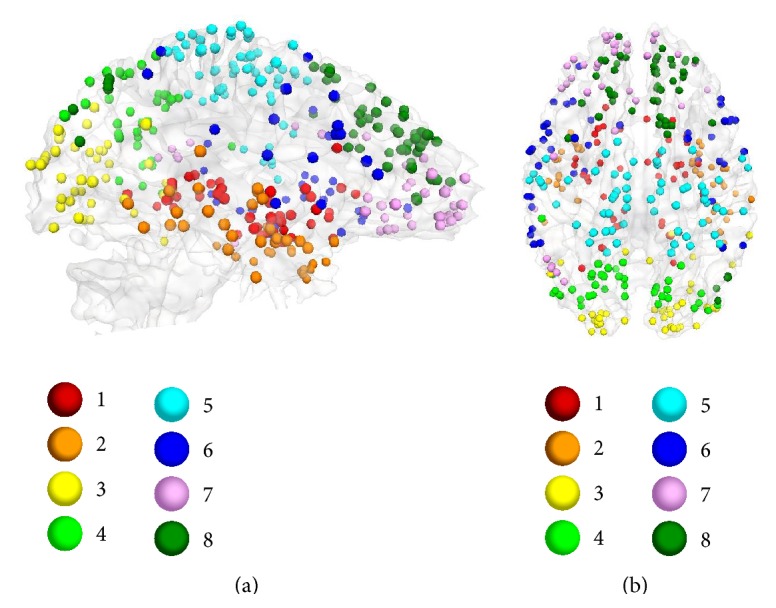
Eight functional connectivity clusters as results of using a multiview group-wise clustering. Eight functional clusters were identified in different colored bubbles.

**Figure 7:**
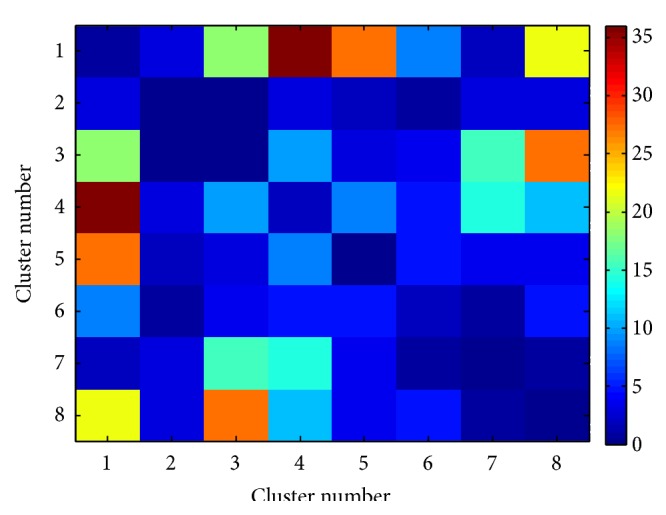
Categorizing the disturbed functional connectivity, which has been obtained using longitudinal statistical analysis by a mixed design ANOVA and NBS, into eight functional connectivity clusters that have been obtained using the multiview group-wise clustering method. Results show that some clusters (especially cluster number 1) are more involved in functional connectivity alteration after mTBI. The color-bar indicates number of involved connectomic signatures.

**Figure 8:**
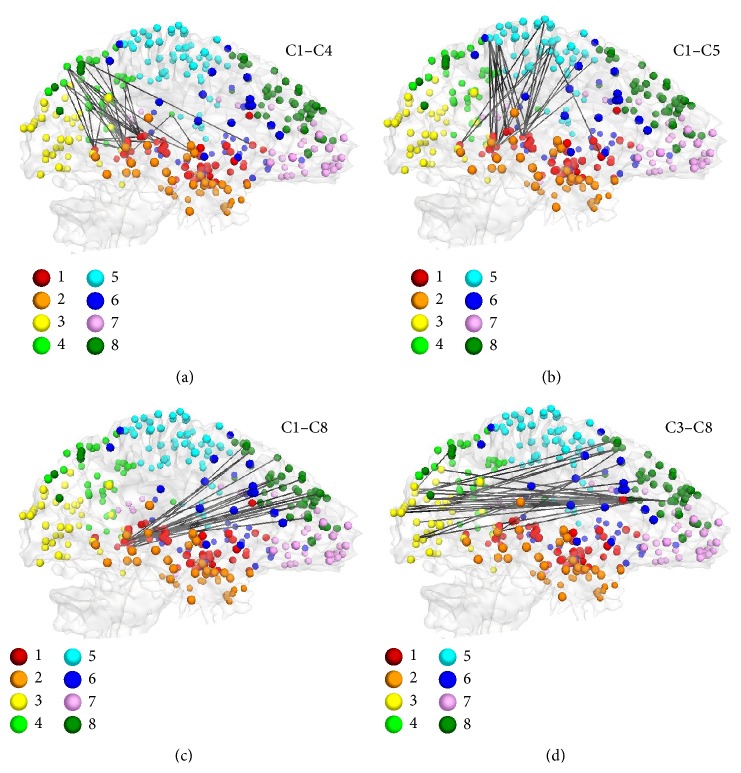
Categorization of affected functional connectivity using the multiview cluster-wise cluster method. (a)–(c) show the connectomic signatures involved in the interaction between cluster number 1 (C1) and clusters numbers 4 (C4), 5 (C5), and 8 (C8), respectively. These interactions represented the important role of the PCC as the central hub of the brain and its interactions with association brain areas as compensatory effects after brain injury. (d) reveals the interaction between the occipital lobe, cluster number 3 (C3), and the frontal lobe, cluster number 8 (C8).

Due to the nature of mTBI and the prevalence of diffuse axonal injury (DAI) pathology in TBI patients, the brain functional response is expected to be seen throughout the brain rather than only in certain restricted regions (see Figure 5). One aim of this study was to discover possible existing patterns among the connectomic signatures after mTBI. For this purpose, we first categorized DICCCOLs into similar clusters based on their rsfMRI time series similarity using a multiview group-wise clustering approach. Using the multiview group-wise clustering approach on all data (a combination of two time points and two groups), we have identified eight functional connectivity clusters (Figure 6). The estimated clusters are similar to the result of our previous work obtained in young healthy subjects [41]. After identifying the corresponding cluster for each DICCCOL, the connectomic signatures were categorized based on the DICCCOLs' clustering information. Results demonstrated that cluster number 1, specifically the posterior cingulate cortex (PCC) portion of it, is the most involved cluster in functional connectivity alterations after mTBI (Figure 7). Interestingly, among all 258 connectomic signatures, 253 (98%) are involved in between-clusters interactions (Figure 7). Further investigation reveals two general patterns among the affected interactions: (I) an increase in functional connectivity between the PCC (from cluster number 1) and the association areas of the brain such as the associative visual cortex, supplementary motor cortex, and the somatosensory association cortex (from clusters numbers 4, 5, and 8), see Figures 8(a)–8(c); and (II) functional hyperconnectivity between the occipital lobe of the brain from cluster number 3 and the frontal lobe area from cluster number 8 (Figure 8(d)).

## 4. Discussion

Our work demonstrates that (a) mTBI affects brain functional connectivity changes at a connectome scale, which is consistent with the published data on diffuse axonal injury pathology [10, 42]. The brain functional connectivity changes at a large scale after mTBI emphasize the necessity of applying connectome-scale study to have a better understanding of brain function and its network substrates of mTBI sequela; (b) there is functional hyperconnectivity across the brain, which can be interpreted as the brain's attempts at large scale to compensate for injury, as has been observed in previous studies [20, 23]; (c) the most affected neurocognitive domains are executive function, attention, and working memory, in line with reported neurocognitive profiles of mTBI patients [43, 44], of which attention and working memory domains are also shown to be particularly susceptible to plasticity in noninjured brains [15, 22, 45, 46]; and (d) the cluster analysis of brain functional connectivity alterations demonstrates two general compensatory patterns: (1) hyperconnectivity between the PCC and the association areas of the brain and (2) increase in frontal-occipital functional connectivity. This study represents a systematic investigation of large-scale brain functional connectivity at resting state in response to mTBI. The results are well consistent with current findings and assumptions of mTBI and validate our hypothesis regarding brain compensation after injury through increasing functional connectivity at a connectome scale, as discussed below.

*Large-Scale Approaches*. Assessment of large-scale brain connectivity is required to properly investigate the brain connectivity alterations after mTBI. Alteration in one connection can directly or indirectly alter other brain connections [7]. The brain is a complex hyperconnected nexus, and any alteration in a brain connection due to mTBI causes a domino effect, which affects other structural or functional connections of the brain. In this situation, the outcome of the brain after injury is not limited to the location and the origin of the injury, and changes in brain functional connectivity are not limited to certain local regions near the site of injury and could be distributed across the brain [11, 13, 47–51], as we already know structural changes like diffuse axonal injury (DAI) to be a common result of mTBI [10, 42, 52]. At the same time, large-scale brain connectivity analyses such as connectome-scale approaches are more sensitive to alterations that are less apparent in gross structure (i.e., white matter integrity and DAI), because large-scale approaches consider each region's integration into the global unit rather than as an independent entity. In light of this, large-scale approaches can provide a better understanding of brain alterations and uncover the role of brain alterations at the macrolevel even in the absence of obvious structural damages [9, 14]. However, to date, most of the brain functional imaging investigations focus on a single or a limited number of brain regions or networks instead of assessing the brain networks at a connectome scale [11, 47, 48, 51], and there is still a lack of investigation into large-scale brain functional connectivity changes after mTBI. Our work supported our hypothesis that mTBI does induce large-scale brain network changes. This is well consistent with the published literature about DAI pathology, which reports multifocal lesions across the brain. It also offers new insights from in vivo imaging perspective. 

*Functional Hyperconnectivity as Compensation*. It has been reported that most mTBI patients suffer reversible brain injury or transient pathophysiological disturbances. After injury, the brain initiates its natural response by cellular repair mechanisms, which leads to recovery, and the brain's compensation through plasticity and modification of brain functional connectivity is a secondary response. Lastly, the brain could experience anatomical plasticity to compensate for the effect of injury [53, 54]. Several studies have shown increases in functional connectivity or hyperconnectivity after different severities of TBI and physical disruption as common brain response [23, 55–57]. Hyperconnectivity was observed mostly in highly connected regions of the brain [55], such as brain regions involved in executive functions and working memory, and central hubs of main brain networks such as the PCC and medial frontal cortex in the default mode network; the dorsolateral prefrontal cortex and parietal cortex in the executive control network; and/or anterior insula in the salience network [53, 55, 58, 59]. Our analysis of whole-brain large-scale networks demonstrates an overall increased functional connectivity in mTBI patients as a brain compensatory response to mTBI. This is consistent with the published literature on separate brain networks [11, 47, 48, 51]. It is of note that our results do not indicate that there was no hypoconnectivity among mTBI group. Our analysis reveals hypoconnectivity in several connections among mTBI patients. However, none of them were detected by the NBS analysis, which only detects disrupted functional connectivity pairs at the network level and does not detect changes if a pair is isolated or not well interconnected with other disrupted pairs in a network. In our data, hypoconnectivity pairs seem to not be well connected enough to survive the NBS analysis. 

*Affected Neurocognitive Domains*. After mTBI, the constellation of clinical and neurocognitive symptoms significantly affects mTBI patients' qualify of life. The detection of neural network substrates of these neurocognitive presentations may help attending physicians order a proper neurorehabilitation program for the patients. Symptoms in several neurocognitive domains have been widely reported in mTBI patients, including attention [56, 60], memory [23, 53, 61–68], processing speed [56], and execution [53, 69]. Among them, attention [56, 60], working memory [23, 56, 61–66], and execution [53, 69] are the most widely reported. Executive function deficits have also been reported in chronic mTBI patients and predicted by DTI data [70]. We hypothesized that the brain functional connectivity alterations should be involved in similar functional and cognitive domains. Categorizing the connectomic signatures using meta-analysis intriguingly validated our hypothesis. The results demonstrate that, among functional and cognitive domains, the interaction between “execution and attention” and “execution and working memory” has been affected the most, which is consistent with the reported patients' symptoms in these neurocognitive domains. Our results further show that the DICCCOLs involved in “action” and “cognition” domains have been involved in brain functional connectivity alterations. This includes the intranetwork connectivity (the connectivity of the DICCCOLs within a cognitive domain) or between-network connectivity (the connectivity between “action” and “cognition” domains). Our study provides the foundation for understanding the changes in functional domains occurring after mTBI, which are similar to changes seen in moderate and severe TBI in adults [56]. 

*Brain Compensatory Patterns*. It has been well reported that the frontal, temporal, and occipital lobes are susceptible to focal contusions due to the direct impact of brain soft tissue onto the rigid inner table of the frontal, occipital, and temporal bones [10, 42]. At the same time, several TBI studies show alterations in functional connectivity of highly connected regions of the brain, specifically the PCC, as a central hub of the brain [11–13, 47, 71]. The multiview group-wise cluster analysis was used to identify the prevalent compensatory pattern of the brain functional connectivity. The results illustrate brain functional connectivity alteration in the expected regions. The PCC demonstrates an increase in functional connectivity with different brain regions including Brodmann area 8 (BA8) of the frontal cortex, the supplementary motor area (BA6), the somatosensory association cortex (BA7), the dorsolateral prefrontal cortex (BA9), the associative visual cortex (BA19), and the anterior cingulate cortex (BA32). Moreover, functional connectivity between the frontal and occipital lobes as two susceptible regions was observed. 

*Limitation and Future Work*. One limitation of this study is the small number of subjects in comparison with the great number of networks and connectivity features, which easily makes the statistical analysis underpowered. A larger sample size and acquiring data at the chronic stage are required to increase the power of statistical analysis and the chance of finding a significant difference. Though we used the NBS approach, a relatively larger number of subjects are indispensable for future investigation to draw a more solid conclusion. Since there is no ground truth of the connectome-scale network alterations, another independent dataset will be necessary in future, as a reproducibility study to cross-validate the findings in the current study.

Another issue is the recovery effect after mTBI. It is expected to see FC recovery in mTBI patients over time. Therefore, assuming similar FC values for a healthy subject and changes in FC for mTBI patient over time, the interaction effect is highly expected. However, our 2×2 design ANOVA does not show statistical significant difference between the two time points of patient group. Although we did not observe a* statistically significant *interaction effect, our analysis shows a trend of recovery in FC of mTBI. Overall functional connectivity of mTBI patients shows a slow trend of approaching the functional connectivity patterns of the healthy subjects over time. Increasing the number of time points would significantly improve a repeated measure data analysis method; thus another data acquisition at the chronic stage is required to evaluate it. It is possible to observe a statistically significant interaction effect if the time gap between the acquisitions was more than 4–6 weeks, allowing for more time to recover and therefore a larger effect size which statistical analysis might have been able to capture. For instance, Nakamura et al. [23] revealed that the overall functional connectivity strength is higher at three months after TBI but that functional connectivity decreases and approaches that of the healthy subjects sample after six months after injury. Moreover, using a larger sample can improve the power of statistical analysis and the chance of finding a significant difference. Furthermore, to apply the current work to clinical management of mTBI patients, the predictive value of the current imaging-based network analysis for mTBI patients' functional and neurocognitive symptoms over long-term recovery is yet to be determined. This could be done by correlating the network-based findings with the patients' chronic neurocognitive and functional assessment, which should also be performed in longitudinal framework.

## 5. Conclusion

In summary, our prospective longitudinal study of mTBI patients at the acute and subacute stages demonstrates functional hyperconnectivity of the brain at a connectome scale. The most affected neurocognitive domains are executive function, attention, and working memory, which is in line with reported neurocognitive profiles of mTBI patients. Furthermore, despite the functional connectivity alterations distributed throughout the whole brain, the cluster analysis reveals two general functional connectivity compensation patterns among mTBI patients, between the PCC and the association areas of the brain and between the occipital and the frontal lobes of the brain.

## Figures and Tables

**Table 1:** Demographic characteristics of healthy controls and patients.

Characteristic	Control subjects	mTBI patients
	(*n* = 24)	(*n* = 16)
Gender		
Male	20	10
Female	4	6
Age (years)		
Mean ± SD	28.00 ± 7.55	34.52 ± 13.85
Median/range	26/(19~50)	30/(19~63)
Race		
African American	2	11
White	15	3
Others	7	2
Cause of injury		
Motorcycle accident	—	1
Bicycle accident	—	1
Motor vehicle accident	—	5
Struck by vehicle	—	2
Assault	—	4
Fall	—	3
Time between injury and 1st scan (hours)		
Mean ± SD	—	82.64 ± 121.90
Median/range	—	17/(3~446)
Time between 2 scans (days)		
Mean ± SD	84.60 ± 55.48	42.68 ± 17.48
Median/range	70/(22~225)	36/(26~89)
Glasgow Coma Scale		
Mean ± SD	—	14.92 ± 0.28
Range	—	(14~15)
